# Necrobiosis Lipoidica Diabeticorum: A Rare and Difficult-to-Treat Condition

**DOI:** 10.7759/cureus.89958

**Published:** 2025-08-13

**Authors:** Binod Kumar, Kiran Kumre, Madhavi Thombare, Tulika Anand, Ratan Kumar

**Affiliations:** 1 Dermatology, Tata Main Hospital, Jamshedpur, IND; 2 Dermatology, Manipal Tata Medical College, Manipal Academy of Higher Education (MAHE), Jamshedpur, IND; 3 Pediatrics, Tata Main Hospital, Jamshedpur, IND; 4 Pediatrics, Manipal Tata Medical College, Manipal Academy of Higher Education (MAHE), Jamshedpur, IND

**Keywords:** collagen, diabetes, granuloma, immunosuppressants, necrobiosis

## Abstract

Necrobiosis lipoidica (NL) is a chronic granulomatous dermatologic condition usually associated with diabetes mellitus (DM). Early diagnosis and treatment can improve the quality of life of the patient and prevent the development of squamous cell carcinoma. Treatment options include corticosteroids, tacrolimus, cyclosporine, fumaric acid esters, biologics (including adalimumab, etanercept, and infliximab), and phototherapy with varying degrees of success. Here, we report a case of NL diabeticorum, which was effectively treated with immunosuppressants and keratolytics.

## Introduction

Necrobiosis lipoidica (NL) is a rare, chronic granulomatous skin condition, commonly affecting the anterior aspect of the legs. It is associated with diabetes mellitus (DM), particularly type 1 DM, and has a predisposition to ulceration. Histopathological features include degeneration of collagen, granulomatous inflammation, and lipid deposition [1]. Trauma to affected areas precipitates ulcer formation. Rarely, long-standing lesions may undergo malignant transformation to squamous cell carcinoma [2-4].

## Case presentation

A 59-year-old female patient visited the dermatology OPD with complaints of multiple thick raised lesions present over bilateral lower limbs for one year. The lesions were itchy and were associated with serous and bloody discharge. The patient had not taken treatment for this condition prior, and was a known case of poorly controlled type II DM. She also had complaints of dyspepsia, tingling, and tenderness over the knee joint, for which she consulted a physician, an orthopedic surgeon, and a neurologist.

**Figure 1:**
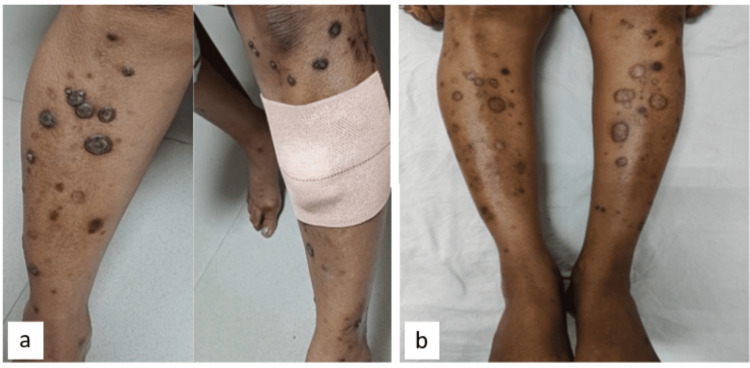
Image of the lesions before (a) and after (b) treatment 1a (before treatment): multiple hyperkeratotic crusted papules and plaques over both lower limbs; 1b (after treatment): most of the crusted plaques have healed, leading to hyperpigmented scars.

On local examination, multiple hyperkeratotic papules, nodules, and ulcerative plaques were present over bilateral lower limbs (Figures 1a, 1b). The lesions started as several well-defined, tiny papules, which further developed into nodules with the passage of time; some of them coalesced to form plaques.

**Figure 2:**
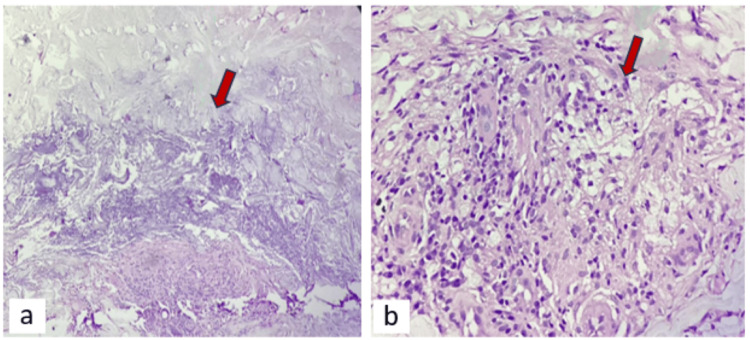
Histopathological examination of the sample from the skin lesion 2a (under low-power microscopy): the arrow shows necrosis with fibrosis; 2b (under high-power microscopy): the arrow indicates granuloma with chronic inflammation.

Skin biopsy was done, which confirmed the condition as NL diabeticorum (Figures 2a, 2b).

The patient was treated with topical steroids, keratolytics, and immunosuppressants (methotrexate) for five months. Oral antibiotics were advised for secondary infection. Symptomatic treatment with antacids and analgesics and treatment for diabetes were continued as per the advice of the physician. All the papules, nodules, and plaques resolved, resulting in hyperpigmented macules and scars. No new lesion has developed after stopping methotrexate over the next six months of follow-up. She continued with topical steroids for post-inflammatory hyperpigmentation. 

## Discussion

NL has a well-recognized but variable association with DM, with over 50% of affected individuals diagnosed with DM. Its incidence among the diabetic population ranges from 0.3% to 1.2% [5]. The temporal association between NL and DM is also variable: NL precedes DM in up to 14% of cases, appears concurrently in 24%, and follows DM in nearly 62% of cases. The development of NL does not appear to correlate with the degree of glycemic control. Other than DM, systemic conditions associated with NL include metabolic syndrome, obesity, hyperlipidemia, hypertension, autoimmune thyroid disease, and celiac disease, suggesting a multifactorial etiopathogenesis [6,7]. 

The cutaneous lesions typically begin as sharply demarcated papules or nodules that progressively merge to form larger plaques. These plaques often display central atrophy, telangiectasia, or a waxy appearance. Although classic features such as a yellow-waxy appearance and telangiectases were absent in our patient, the lesions were progressing, leading to cosmetic concern for the patient. There was an excellent response to methotrexate in the index case, which was prescribed for five months. Contrary to the usual chronic and treatment-resistant course of the disease, no recurrence was seen during a six-month follow-up. This underscores the multifaceted nature of NL and the evolving therapeutic landscape driven by emerging research. 

## Conclusions

Treatment for NL remains challenging. The course of disease is chronic with variable progression and scarring. In longstanding lesions, particularly those that have undergone repeated trauma or ulceration, there is a risk of malignant transformation to squamous cell carcinoma. Ulcerations in lesions can cause significant morbidity, as they are frequently painful, susceptible to infection, and often require extended periods of wound care. The primary goal of treatment is to limit the progression of existing lesions and to prevent complications.
